# CD47 is an adverse prognostic factor and a therapeutic target in gastric cancer

**DOI:** 10.1002/cam4.478

**Published:** 2015-06-16

**Authors:** Kazumichi Yoshida, Hironori Tsujimoto, Kouji Matsumura, Manabu Kinoshita, Risa Takahata, Yusuke Matsumoto, Shuichi Hiraki, Satoshi Ono, Shuhji Seki, Junji Yamamoto, Kazuo Hase

**Affiliations:** 1 Department of Surgery, National Defense Medical CollegeTokorozawa, Saitama, Japan; 2 Laboratory Center, National Defense Medical CollegeTokorozawa, Saitama, Japan; 3 Department of Immunology and microbiology, National Defense Medical CollegeTokorozawa, Saitama, Japan; 4 Division of Traumatology, National Defense Medical CollegeTokorozawa, Saitama, Japan

**Keywords:** CD44, CD47, gastric cancer, phagocytosis

## Abstract

CD47 is an antiphagocytic molecule that acts via ligation to signal regulatory protein alpha on phagocytes; its enhanced expression and therapeutic targeting have recently been reported for several malignancies. However, CD47 expression in gastric cancer is not well documented. Immunohistochemical expression of CD47 in surgical specimens was investigated. Expression of CD47 and CD44, a known gastric cancer stem cell marker, were investigated in gastric cancer cell lines by flow cytometry. MKN45 and MKN74 gastric cancer cells were sorted by fluorescence-activated cell sorting according to CD44 and CD47 expression levels, and their in vitro proliferation, spheroid-forming capacity, and in vivo tumorigenicity were studied. In vitro phagocytosis of cancer cells by human macrophages in the presence of a CD47 blocking monoclonal antibody (B6H12) and the survival of immunodeficient mice intraperitoneally engrafted with MKN45 cells and B6H12 were compared to experiments using control antibodies. Immunohistochemistry of the clinical specimens indicated that CD47 was positive in 57 out of 115 cases, and its positivity was an independent adverse prognostic factor. Approximately 90% of the MKN45 and MKN74 cells expressed CD47 and CD44. CD47^hi^ gastric cancer cells showed significantly higher proliferation and spheroid colony formation than CD47^lo^, and CD44^hi^CD47^hi^ cells showed the highest proliferation in vitro and tumorigenicity in vivo. B6H12 significantly enhanced in vitro phagocytosis of cancer cells by human macrophages and prolonged the survival of intraperitoneal cancer dissemination in mice compared to control antibodies. In conclusion, CD47 is an adverse prognostic factor and promising therapeutic target in gastric cancer.

## Introduction

Gastric cancer is the fourth most common type of cancer and the second most frequent cause of cancer-related deaths, accounting for ∼10% of cancer deaths worldwide 1,2. The etiology of gastric cancer has been completely obscure for many decades. However, several considerable advances in the knowledge of the carcinogenesis of gastric cancer and the development of novel anticancer agents have recently been made.

Recent cancer stem cell (CSC) models suggest that in many malignancies, tumor initiation and propagation are driven by a small population of self-renewing tumor cells 3. CSCs also promote tumor cell heterogeneity, metastasis, and therapeutic resistance 4. The study of CSCs would be greatly enhanced by the availability of specific markers including ALDH1, CD133, CD44, CD24, CD166, EpCAM, and CD47 to identify and isolate these cells 5–10. The purification and characterization of CSCs could lead to the identification of better targets for therapeutic interventions. This new paradigm has remarkable implications for cancer therapy because current therapies are more successful at eradicating non-CSCs than CSCs 11–14.

In gastric cancer, CD44 has been reported to be a useful CSC marker and its expression is correlated with enhanced tumorigenicity, chemoradioresistance, 8. and adverse prognosis 12,13. Moreover, according to recent reports, a CD44 variant suppresses the accumulation of reactive oxygen species in gastric cancer cells by stabilizing the glutamate-cystine transporter and controlling the intracellular level of glutathione 14. This defense mechanism against oxidative stress partly accounts for the enhanced tumorigenicity and chemoradioresistance of CD44-positive gastric cancer cells.

CD47, which is also termed integrin-associated protein, is a 50-kDa cell surface glycoprotein that serves as an antiphagocytic molecule via binding to signal regulatory protein alpha (SIRP*α*), which is expressed on various phagocytic cells such as dendritic cells and macrophages 15,16. Thus, CD47 expression helps cells evade innate immunity. However, CD47 is broadly expressed on hematopoietic cells and other normal tissues 17, and the enhanced expression of CD47 has been reported on tumor cells in a variety of hematopoietic malignancies and solid tumors, such as leukemia 6, bladder cancer 18, astrocytoma 19, prostate cancer 20, and leiomyosarcoma 21. CD47 is known to have pleiotropic functions. For instance, CD47 monoclonal antibody induces caspase-independent leukemia cell death in chronic lymphocytic leukemia 22. In addition, CD47 stimulation induces cancer cell proliferation via a PI3K/Akt-dependent pathway in astrocytoma 19, and is also known to be associated with angiogenesis via vascular endothelial growth factor receptor-2 23. Thus, CD47 is associated with tumor progression, metastasis, and outcome, which suggests the CSC-based therapeutic potential of targeting CD47 in cancer 7,20,21,24–26. However, little is known regarding the expression of CD47, which may be associated with tumor progression in gastric cancer.

In this study, we investigated the expression of CD47 in gastric cancer in clinical and experimental settings, and we elucidated the therapeutic potential of targeting CD47 for treating gastric cancer.

## Materials and Methods

### CD47 expression in gastric cancer tissue

In a clinical setting, 115 serial gastric cancer cases with primary tumors that were deeper than submucosal invasion (T1b) without any distant metastases, and who underwent potentially curative gastrectomy between 1995 and 1997 at the National Defense Medical College Hospital (Tokorozawa, Saitama, Japan), were included in this study. Surgical specimens were prepared in a formalin-fixed paraffin-embedded tissue microarray setting in which at least two specimens were obtained from the periphery and center of the primary tumor for each case. Four-micrometer-thick sections were cut from formalin-fixed, paraffin-embedded blocks and mounted on silane-coated glass slides. After dewaxing and rehydration with dH_2_O, the sections for immunostaining were autoclaved for antigen retrieval (120°C, 10 min) in a target retrieval solution at pH 9.0 (Dako Japan, Tokyo, Japan). After cooling, nonspecific antibody binding was inhibited by incubating the sections in 4% skim milk. Endogenous peroxidase activity was blocked by using 5% H_2_O_2_. After transfer to a humidified chamber, the sections were incubated with anti-CD47 monoclonal antibody (clone B6H12; LifeSpan Bioscience, Inc., Seattle WA) at a dilution of 1:12.5 (final concentration of 40 *μ*g/mL) at 4°C overnight. Subsequently, the sections were incubated with peroxidase-labeled polymer (EnVisionTM + System-HRP; Dako Japan) for 120 min at room temperature. For antigen visualization, sections were immersed in 0.05% diaminobenzidine tetrahydrochloride solution containing 0.01% hydrogen peroxidase for 10 min and counterstained lightly with Mayer's hematoxylin. The cytoplasmic membranous staining of CD47 on cancer cells was evaluated for the most evident staining area of the microscopic field at 200× magnification. For more than 50% of the cancer cells, faint or barely perceptible cytoplasmic staining was graded as being “very weak”, weak cytoplasmic staining as “weak”, cytoplasmic and membranous staining as “moderate”, and strong cytoplasmic and membranous staining as “strong”. A grade of more than moderate was considered to indicate the positive expression of CD47.

The clinical stage of each gastric cancer was assigned according to the 7th edition of the International Union Against Cancer's TNM classification of malignant tumors 27.

### Cell culturing

Human gastric cancer cell lines (MKN7, MKN45, MKN74, KATOIII, and NUGC3) were purchased from the Riken Bioresource Center (Cell Bank, Ibaraki, Japan) and the Japanese Collection of Research Bioresources (JCRB Cell Bank, Osaka, Japan). All cell lines were cultured in culture medium (RPMI 1640 medium [Sigma-Aldrich, St. Louis, MO] containing 10% heat-inactivated fetal bovine serum [FBS, Life Technologies, Carlsbad, CA], 100 U/mL of penicillin, 100 *μ*g/mL of streptomycin, and 0.25 *μ*g/mL of amphotericin B [Antibiotic-Antimycotic; Life Technologies]) at 37°C in 5% CO_2_ with 95% humidity.

### Flow cytometric analysis and FACS

Semi-confluent (70–90%) gastric cancer cells in a 100-mm tissue culture dish were washed with phosphate-buffered saline (PBS; Dulbecco's phosphate-buffered saline, Sigma-Aldrich), dissociated from the dish by using 0.25% Trypsin-EDTA (Life Technologies), and centrifuged at 126*g* for 5 min. Subsequently, the cell pellets were resuspended with Hanks' balanced salt solutions (HBSS, Life Technologies) containing 10 mmol/L *N*-2-hydroxyethylpiperazine-*N*′-2-ethanesulfonic acid buffer and 3% FBS, and incubated for 30 min at room temperature with 100-fold diluted antibodies at final concentrations of 1% (w/v). Fluorescein isothiocyanate (FITC)-conjugated mouse anti-human CD47 (clone B6H12; eBioscience, San Diego CA), FITC- or Phycoerythrin (PE)-conjugated rat anti-human/mouse CD44 (clone IM7; eBioscience), FITC-conjugated mouse anti-human HLA-ABC (clone W6/32; eBioscience), FITC- or PE-conjugated mouse IgG1*κ* (eBioscience) were used for primary antibodies. After incubation with antibody for 30 min, the cells were resuspended with 3% FBS-containing HBSS and flow cytometric analysis was performed by using a BD FACSCalibur flow cytometer (BD Biosciences, San Jose, CA). Propidium iodide (PI) was used to exclude dead cells. Fluorescence-activated cell sorting (FACS) was performed by using a BD FACSVantage SE cell sorter (BD Biosciences). The results were analyzed by using Flowjo software (Tree Star, Inc., Ashland OR). The estimated accuracy of the cell sorting was over 95%.

The highest and lowest 20% of CD47 expressers out of the whole-cell population were defined as being CD47^hi^ and CD47^lo^, respectively. CD44^hi^ and CD44^lo^ were defined in the same manner.

### Spheroid colony assay

CD47^hi^ or CD47^lo^ gastric cancer cells were cultured in each well of a 96-well ultra-low attachment tissue culture plate (Corning Life Science, Acton, MA) at a density of 20 cells/well with 200 *μ*L of serum-free RPMI-1640 containing 20 ng/mL of human recombinant epidermal growth factor (Life Technologies) and 10 ng/mL of human recombinant basic fibroblast growth factor (Life Technologies). After 3 week of culturing, images of the spheroid colony in each well were recorded using a microscope (BZ-8000; Keyence, Osaka, Japan). Each recorded image was analyzed to measure the area of the spheroid colony using ImageJ software (U.S. National Institutes of Health, Bethesda, MD; http://rsb.info.nih.gov/ij/) according to the description of the area measurements of a complex object in the instruction manual. This experiment was performed in triplicate.

### Proliferation assay

The sorted cells were incubated in each well of a flat-bottomed 96-well tissue culture plate (BD Biosciences) at a density of 100 cells/well (10 wells per fraction). Each well was supplied with 200 *μ*L of culture medium. Medium with or without the indicated antibody was replaced every 3 days. Two week after incubation, the viable cells were estimated using a Cell Counting Kit-8 (Dojindo Laboratories, Tokyo, Japan) according to the manufacturer's protocol. Briefly, the optical absorbance of each well at a wavelength of 450 nm was measured to estimate the production of formazan, which reflects the number of viable tumor cells, by using a SpectraMax Plus 384 Microplate Spectrophotometer (Molecular Devices Japan, Tokyo, Japan). This experiment was performed in triplicate.

### Tumorigenicity assay in severe combined immunodeficiency mice

Eight-week-old male C.B-17/lcr-*scid/scid*Jcl severe combined immunodeficiency (SCID) mice were purchased from the Central Laboratories for Experimental Animals Japan, Inc. (CLEA Japan Inc., Tokyo, Japan). Gastric cancer cells (1 × 10^5^) suspended in 50 *μ*L of medium and 50 *μ*L of growth factor reduced Matrigel (BD Biosciences) were inoculated into the dorsal subcutaneous layer. Eight weeks after inoculation, the mice were sacrificed and the tumors were harvested. The weight and size of each tumor was measured. This experiment was independently repeated five times for each cell line.

### Apoptosis of gastric cancer cells induced by anti-CD47 antibody

Forty-eight hours after incubation with CD47 blocking monoclonal antibody (B6H12; eBioscience) or mouse IgG1*κ* antibody (eBioscience), apoptosis of the gastric cancer cells was evaluated by flow cytometry using an Annexin V Apoptosis Detection Kit (BioVision, Milpitas CA) according to the manufacturer's protocol. The PI-negative Annexin V-positive cell fraction was defined as comprising apoptotic cells. This experiment was performed in triplicate.

### Phagocytosis assay with human and murine macrophages

For human peripheral blood monocyte (PBMC) separation, 30 mL of fresh human blood was taken from healthy volunteers. The blood samples were processed according to a density gradient centrifugation method using Lymphocyte Separation Medium (MP Biomedicals Japan, Tokyo, Japan) to obtain a leukocyte-enriched white buffy coat.

To obtain murine bone marrow cells (BMCs), the femurs were aseptically removed from 8-week-old Balb/c mice and both ends of the bone were cut off. The bone marrow of each femur was flushed with cold PBS through a 27-gauge needle into a conical tube. The tube was centrifuged at 128*g* for 5 min and the cell pellet was resuspended in RPMI1640 medium.

For obtaining macrophages, a previously reported standard protocol was employed 28–30. A total of 5 × 10^7^ human PBMCs or murine BMCs were plated on a poly-D-lysine-coated 100-mm dish (Biocoat, BD Biosciences) with 10 mL of RPMI1640 containing 10% FBS (culture medium) and incubated for 2 h. The supernatant with nonadherent cells was then removed and washed with PBS. Human recombinant monocyte colony-stimulating factor (eBiosciences) at 50 ng/mL in 10 mL of culture medium was added and the cells were cultured for 7 days. The culture media was replaced every 3 days. Seven days after culturing, the medium was removed and the adherent cells were washed with PBS. Subsequently, 1 mL of 0.25% Trypsin/EDTA solution was added and the suspension, which was then incubated for 30 min at room temperature with gentle tipping of the dish to dissociate the macrophages. The cell suspension was centrifuged at 126*g* for 5 min.

The PBMC- or BMC-derived macrophages were fluorescently labeled with a PKH67GL green fluorescent cell linker kit (Sigma-Aldrich) according to the manufacturer's instructions. Similarly, human gastric cancer cells were labeled with a PKH26GL red fluorescent cell linker kit. PKH64GL-labeled macrophages were plated in the wells of a 24-well tissue culture plate at a density of 5 × 10^4^ cells/well and incubated for 6 h to allow the macrophages to attach to the bottoms of the wells. Then, 2 × 10^5^ PKH26GL-labeled gastric cancer cells were added with 10 *μ*g/mL IgG1 isotype, HLA-ABC, or B6H12 antibody, and they were cultured for 2 h. After being washed repeatedly with warm PBS, the cells were fixed with 0.4% paraformaldehyde. The phagocytic index was counted under the fluorescent microscope (BZ-8000; Keyence) as the number of red phagocytosed gastric cancer cells per 100 green macrophages in randomly selected microscopic fields. The experiments that involved using human PBMC-derived and murine BMC-derived macrophages were independently repeated five times using peripheral blood from five different healthy donors and four times using bone marrow from four different mice, respectively.

### Therapeutic effect of anti-CD47 antibody in the intraperitoneal dissemination model

Six-week-old male nude mice (BALB/cAJcl-nu/nu) were purchased from CLEA Japan, Inc. At 8-week old, the animals were intraperitoneally injected with 5 × 10^6^ MKN45 cells in 500 *μ*L of PBS with 100 *μ*g of anti-CD47, anti-HLA-ABC, and IgG1 isotype antibody, and their survival and body weights were monitored. Each group consisted of 10 mice (*n* = 10).

### Statistical analysis

The data are represented as the mean ± standard deviation. Statistical analysis was performed by using the two-tailed Student's *t*-test for the comparison of two groups or standard one-way analysis of variance followed by the post hoc Tukey's test for comparing more than three groups. Survival curves were generated by using the Kaplan–Meier method and the significance of the difference in survival was determined by employing the log-rank test. The Cox proportional hazard model was employed for univariate and multivariate survival analyses. All analyses were performed by using JMP10 software (SAS Institute Japan, Tokyo, Japan) and significant differences are represented throughout as *P *<* *0.05.

### Ethics

All of the animal studies were approved by the Ethical Board of the Center for Laboratory Animal Science at the National Defense Medical College and conducted according to the Guidelines for the Care and Use of Laboratory Animals. For immunohistochemistry, informed consent in written form was obtained from all individuals prior to surgery. All studies concerning human specimens were approved by the Institutional Review Board of the National Defense Medical College.

## Results

### Immunohistochemical detection of CD47 in clinical gastric cancer specimens

**Figure 1:**
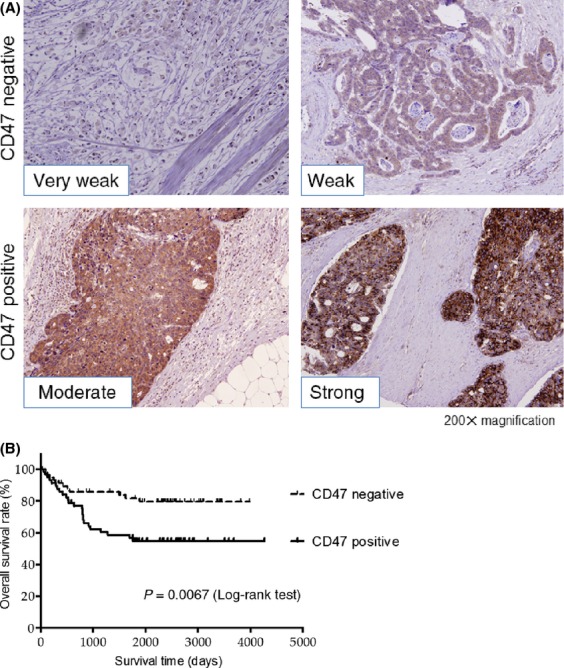
(A) Representative image of CD47-positive and CD47-negative gastric cancer. (B) Overall survival after curative resection of gastric cancer. The overall 5-y survival rate was 54.8% for CD47-positive cases and 79.9% for CD47-negative cases, respectively. CD47 positivity of the primary tumor as observed by immunohistochemistry was correlated with a poor prognosis.

Representative resected gastric cancer specimens that were immunohistochemically positive and negative for CD47 are depicted in Figure1A. We observed positive expression of CD47 in the tumor cells of 57 out of 115 patients (49.5%), and no significant differences in clinicopathological characteristics between the CD47-positive and CD47-negative cases were observed, except for the tumor locations (data not shown).

The overall survival rate for the CD47-positive cases was significantly lower than that for the CD47-negative cases (Fig.1B). Univariate analysis of clinicopathological factors associated with overall survival revealed the age, tumor location, tumor size, tumor depth (T factor), nodal involvement (N factor), and CD47 staining, while the multivariate analysis demonstrated that age, tumor size, nodal involvement, and CD47 staining were significantly independent prognostic factors in this cohort (Table1).

**Table 1:** Univariate and multivariate analysis of clinicopathological factors for overall survival

	Univariate analysis			Multivariate analysis		
	HR	95% CI	*P*-value	HR	95% CI	*P*-value
Age (60 <=)	1.86	0.92–4.06	0.081			
Gender (male)	1.30	0.64–2.83	0.47			
Tumor location (U area)	2.54	1.27–4.93	0.0094	2.17	1.00–4.58	0.049
Tumor size (5 cm <=)	3.81	1.91–8.10	0.0001	2.88	1.37–6.42	0.0048
Histological type (diffuse)	1.76	0.91–3.52	0.093			
Stroma (Schirrous)	1.50	0.72–2.94	0.26			
T factor (T3 <=)	4.36	2.07–10.28	<0.0001	2.01	0.82–5.44	0.12
N factor (N1 <=)	5.66	2.40–16.60	<0.0001	3.32	1.25–10.54	0.0138
CD47-positive	2.57	1.29–5.46	0.0062	2.35	1.11–5.27	0.024

CD47 positivity, as observed by immunohistochemistry analysis of resected specimens, was an independent adverse prognostic factor along with tumor location, tumor size, and lymph node metastasis (N factor) as determined by multivariate analysis. HR, hazard ratio.

### CD47 and CD44 expression in gastric cancer cell lines

**Figure 2:**
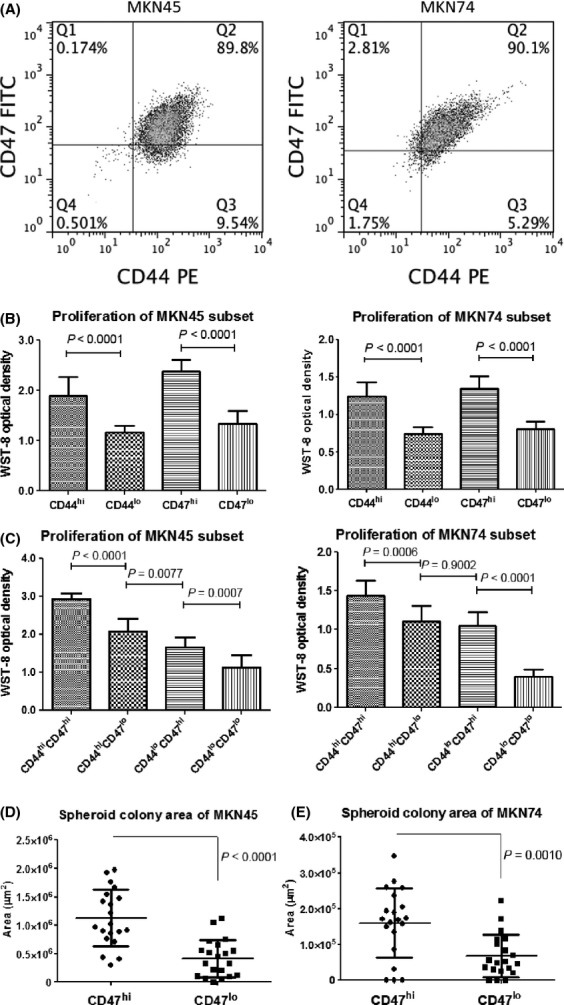
Proliferation and spheroid colony formation of CD47-expressing gastric cancer cells. (A) MKN45 and MKN74 gastric cancer cell lines, for which ∼90% of the tumor cells were positive for CD44 and CD47. (B and C) Cancer cell proliferation of the indicated fraction was estimated by using a WST-8 cell proliferation assay. The WST-8 optical density reflects the number of viable tumor cells. CD44^hi^ and CD47^hi^ gastric cancer cells showed a higher proliferation compared with their low-level counterparts (B). High and low expression of both cell surface markers was correlated with the highest and lowest rates of proliferation, respectively (C). (D) and (E) CD47^hi^ gastric cancer cells formed significantly larger spheroid colonies compared with CD47^lo^ gastric cancer cells under the nonadherent serum-free condition, which suggests that the CD47^hi^ fraction had cancer-initiating capacity.

Flow cytometric analyses revealed that the MKN45 and MKN74 gastric cancer cell lines expressed both CD44 and CD47 on ∼90% of the tumor cells (Fig.2A). Similarly, the other gastric cancer cell lines, such as MKN7, KATOIII, and NUGC3, expressed both CD44 and CD47 with different fluorescence intensities (data not shown).

### Spheroid colony formation of gastric cancer cells expressing CD47

Since spheroid colony formation in serum-free medium under nonadherent conditions was considered to be a typical approach to surveying candidates for CSCs in vitro 8,31, we performed a spheroid colony assay to evaluate the spheroid-forming ability of CD47^hi^ gastric cancer cells in comparison with CD47^lo^. However, both the CD47^hi^ and CD47^lo^ gastric cancer cells formed small clusters in a few weeks: CD47^hi^ cancer cells showed sustained spheroid growth for a longer time than did the CD47^lo^ cancer cells. Consequently, the CD47^hi^ gastric cancer cells formed significantly larger spheroid colonies than their CD47^lo^ counterparts for the MKN45 and MKN74 gastric cancer cell lines (Fig.2D).

### In vitro proliferation of gastric cancer cells expressing CD44 and CD47

We evaluated the proliferation of gastric cancer cells according to the expression levels of CD44 and CD47, that is, CD44^hi^ or CD44^lo^ and CD47^hi^ or CD47^lo^. The proliferation of the CD44^hi^ gastric cancer cells was significantly higher than that of their CD44^lo^ counterparts in both gastric cancer cell lines (Fig.2A). Similarly, proliferation of the CD47^hi^ gastric cancer cells was significantly higher than that of their CD47^lo^ counterparts (Fig.2B). Specifically, the CD44^hi^CD47^hi^ cell fraction exhibited the highest proliferation capacity compared with their counterparts, and the CD44^lo^CD47^lo^ population exhibited the lowest proliferation in both cell lines (Fig.2C).

### CD44^hi^CD47^hi^ gastric cancer cells show enhanced tumorigenicity in vivo

**Figure 3:**
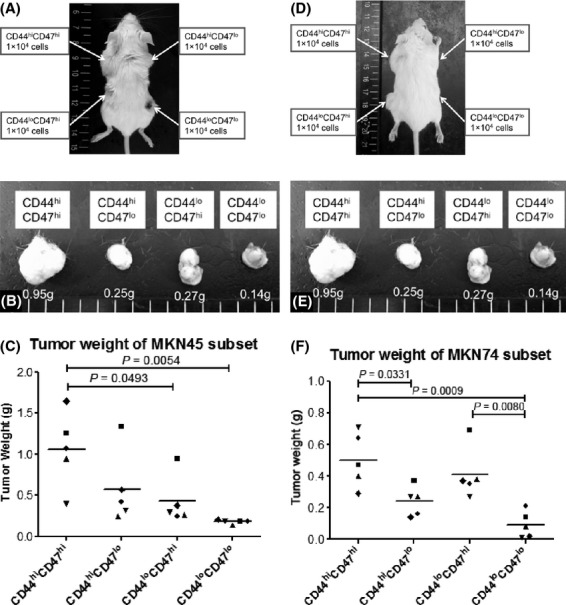
In vivo tumorigenicity in SCID mice. (A) Eight weeks after MKN45 cell inoculation, this SCID mouse developed tumors. (B) Resected tumors that were produced from MKN45 gastric cancer cells. (C) The tumor weight of the MKN45 subset. The different symbols on the plot represent tumors that formed in a different mouse of an independent experiment. The mean is indicated by the horizontal bar. (D) Eight weeks after MKN74 gastric cancer cell inoculation. (E) Resected tumors of MKN74 gastric cancer cells. (F) The tumor weight of the MKN74 gastric cancer cell subset.

We performed xenograft transplantations of FACS-sorted gastric cancer cells according to the CD44 and CD47 expression levels of the MKN45 and MKN74 cancer cell lines into the subcutaneous space of SCID mice. Three to 4 weeks after inoculation, only the CD44^hi^CD47^hi^ gastric cancer cell fraction developed palpable tumors. Eight weeks thereafter, the CD44^hi^CD47^hi^ cells generated the largest and heaviest tumor compared with their counterparts, and the CD44^lo^CD47^lo^ population formed the smallest tumors for both cell lines (Fig.3A and B). All of the tumors were pathologically confirmed as being carcinomas on formalin-fixed, paraffin-embedded sections with hematoxylin and eosin staining (data not shown).

### Effect of anti-CD47 antibody on gastric cancer cell proliferation and apoptosis

In order to investigate the effect of the B6H12 antibody on the proliferation and apoptosis of gastric cancer cells in vitro, CD47^hi^ and CD47^lo^ cells were incubated with B6H12 antibody or an isotype control at serial concentrations. The proliferation of both CD47^hi^ and CD47^lo^ cells was unaffected by the B6H12 antibody for both the MKN45 and MKN74 gastric cancer cell lines (data not shown). In addition, we did not find any difference in the incidence of apoptotic cells between incubation with the B6H12 antibody and the isotype control for both cell lines (data not shown).

### Effect of anti-CD47 antibody on phagocytosis of gastric cancer cells by human PBMC-derived macrophages

**Figure 4:**
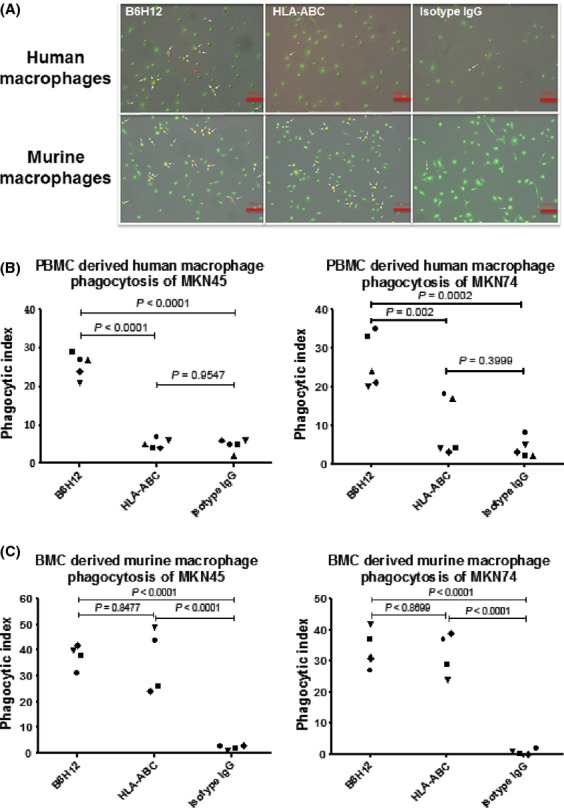
B6H12 antibody promoted human and murine macrophage phagocytosis of cancer cells in vitro. (A) Representative image of MKN45 gastric cancer cells (red) phagocytosed by peripheral blood monocyte-derived human macrophages (green) (upper panels) and bone marrow cell-derived murine macrophages (green) (lower panels). The arrows indicate phagocytosed gastric cancer cells. Red bars indicate 100 *μ*m. (B and C) Phagocytic index of gastric cancer cells ingested by peripheral blood monocyte-derived human macrophages (B) and bone marrow cell-derived murine macrophages (C).

In order to determine whether treatment with the B6H12 antibody might enhance the phagocytosis of gastric cancer cells by macrophages, gastric cancer cells were cultured with PBMC-derived human macrophages in the presence of B6H12, HLA-ABC, or an isotype control. The B6H12 antibody significantly enhanced human macrophage phagocytosis of gastric cancer cells compared with the HLA-ABC antibody or the isotype control (Fig.4A and B). On the other hand, both B6H12 and HLA-ABC antibody significantly facilitated the phagocytosis of gastric cancer cells by murine macrophages compared with isotype IgG (Fig.4A and C).

### Anti-CD47 antibody improves the survival rate in an intraperitoneal cancer dissemination model

**Figure 5:**
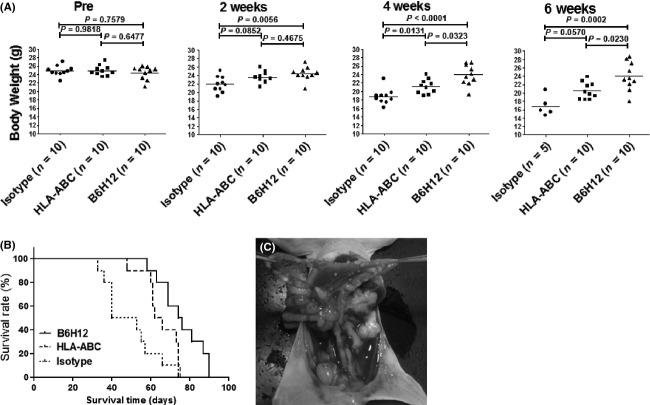
Therapeutic effect of anti-CD47 antibody in a MKN45 peritoneal dissemination mouse model. (A) Mice treated with B6H12 antibody sustained heavier body weights compared with their counterparts from 4–6 week after cancer engraftment. The mean ± SD is indicated by the horizontal bar. (B) Survival curves of mice treated with 100 *μ*g of B6H12 (*n* = 10), HLA-ABC (*n* = 10), or mouse IgG1 isotype antibody (*n* = 10). The B6H12 antibody significantly prolonged survival, even compared with the HLA-ABC antibody. Isotype versus HLA-ABC,*P *=* *0.0246; isotype versus B6H12, *P *=* *0.0003; HLA-ABC versus B6H12, *P *=* *0.0187. (C) Laparotomy findings of an intraperitoneal dissemination mouse model. Disseminated cancer nodules were formed on the peritoneal surfaces of the intestine and mesentery.

To assess the in vivo therapeutic effect of blocking CD47, MKN45 gastric cancer cells that were treated with B6H12, HLA-ABC, or an isotype control were intraperitoneally injected into nude mice, and the mice were then monitored in terms of their body weights and survival rate. The average body weight of the mice in each group gradually decreased throughout the investigation. At 3–6 week after injection, the mice that were injected with B6H12-treated cells had heavier body weights than those that were injected with HLA-ABC-treated or isotype IgG-treated cells (Fig.5A). For the survival analysis, although both the B6H12 and HLA-ABC antibodies significantly prolonged survival compared with the isotype IgG, the mice that were injected with B6H12-treated cells survived for a significantly longer time compared with those that were with injected with HLA-ABC-treated cells (Fig.5B). Laparotomy findings of the intraperitoneal dissemination of a mouse are shown in Figure5C.

## Discussion

In this study, we demonstrated that patients with highly expressed CD47 in gastric cancer tissue had unfavorable outcomes and that CD47 expression on gastric cancer cells was an independent prognostic factor. Furthermore, CD44^hi^CD47^hi^ gastric cancer cells exhibited enhanced proliferation both in vitro and in vivo. We also showed that a CD47 blocking antibody enhanced the phagocytosis of gastric cancer cells by macrophages and significantly improved the survival rate in an intraperitoneal cancer dissemination model.

CD47 serves as the ligand of SIRP*α*, which is expressed on phagocytic cells and, when activated, initiates a signal transduction cascade resulting in the inhibition of phagocytosis 32–34. Majeti et al. demonstrated that increased CD47 expression in acute myeloid leukemia cells is associated with a poor clinical outcome, and that the blocking of CD47 enables the phagocytosis of tumor cells by macrophages 6. In this study, we demonstrated that a blocking antibody directed against CD47 preferentially enabled the phagocytosis of gastric cancer cells by human macrophages as well as murine macrophages. In addition, our study showed that an HLA-ABC antibody also facilitated the phagocytosis of human gastric cancer cells by murine macrophages. The enhanced phagocytosis by murine macrophages might be associated with antibody opsonization or antibody-dependent cellular cytotoxicity (ADCC), which facilitates phagocytosis 35. It has been reported that the human Fc receptor does not bind or binds poorly to the Fc portion of the murine IgG1 antibody, whereas the murine Fc receptor binds to the Fc portion of both human and murine antibodies 36,37. Thus, the enhanced phagocytosis of human cancer cells by human macrophages could be mediated by the blockade of CD47-SIRP*α* signaling, but not in an Fc-mediated fashion.

We also showed that CD47^hi^ gastric cancer cells, especially CD44^hi^CD47^hi^ cells, exhibit extremely enhanced proliferation in vitro and enhanced tumorigenicity in vivo. Furthermore, the CD47 blocking antibody improved the survival rate compared with control antibodies in our xenograft peritoneal dissemination model. CD47 blocking antibodies have been shown to directly induce apoptosis and to influence tumor proliferation in several malignant cell lines 22,38. However, a B6H12 antibody did not affect the apoptosis and proliferation of gastric cancer cells in this study. Thus, CD47 blocking antibodies may have different effects depending on the type of tumor that it targets.

In our study, ∼90% of the MKN45 and MKN47 gastric cancer cells highly co-expressed CD44 and CD47. In clinical settings, CD44 expression has been reported to be an unfavorable prognostic factor in gastric cancer patients 5,39. Takaishi et al. demonstrated that CD44-positive gastric cancer cells have the ability to undergo spheroid colony formation in vitro, exhibit tumorigenicity in immunodeficient mice, and possess characteristics that are reminiscent of stem cells, including self-renewal and the potential to aberrantly differentiate into non-CSC and initiate tumor formation 8. However, CD44 is known to be a receptor for hyaluronic acid, with over 800 CD44 variants. It also has more than 20 distinct isoforms 40, each one of which is distinctively expressed under different conditions, which leads to difficulty in clinical applications of CD44-targeted therapy for cancer treatment. CD47 is also broadly expressed on hematopoietic cells and other normal tissues 41, which could potentially lead to toxic effects from anti-CD47 antibody therapy. However, it has been reported that the administration of anti-mouse CD47 antibody to wild-type mice causes modest toxicity, principally isolated neutropenia 6. In this regard, CD47-targeted therapy may be advantageous for clinical applications to treat gastric cancer as a CSC-based therapy.

In conclusion, both CD44 and CD47 were highly expressed in gastric cancer cells, and their higher expressions correlated with higher proliferation and tumorigenicity in vitro and in vivo. In addition, blocking CD47 signaling induced the phagocytosis of tumor cells by macrophages, which suggests that CD47 expression on tumor cells may help tumors to evade immunosurveillance in their hosts. Therefore, targeting CD47 should be a promising and fruitful therapeutic approach for treating gastric cancer. A more detailed understanding of the modulation of CD47 could open new avenues for CSC-based immunotherapy for treating gastric cancer.

## Conflict of Interest

None declared.
